# Efficacy of Segmental Muscle Vibration on Pain Modulation in Patients with Primary Cervical Dystonia Treated with Botulinum Type-A Toxin: A Protocol for a Randomized Controlled Trial

**DOI:** 10.3390/neurosci6020030

**Published:** 2025-04-02

**Authors:** Riccardo Buraschi, Paolo Pedersini, Giacomo Redegalli, Rosa Pullara, Joel Pollet, Marina Rossi, Massimiliano Gobbo, Sara Gueli, Maurizio Falso

**Affiliations:** 1 IRCCS Fondazione Don Carlo Gnocchi, Milan, Italy; rburaschi@dongnocchi.it (R.B.); gredegalli@dongnocchi.it (G.R.); rpullara@dongnocchi.it (R.P.); jpollet@dongnocchi.it (J.P.); marirossi@dongnocchi.it (M.R.); massimiliano.gobbo@unibs.it (M.G.); sgueli@dongnocchi.it (S.G.); mfalso@dongnocchi.it (M.F.); 2 Department of Clinical and Experimental Sciences, University of Brescia, Brescia, Italy

**Keywords:** cervical dystonia, segmental muscle vibration, pain management, rehabilitation

## Abstract

Primary cervical dystonia (PCD), or spasmodic torticollis, is a focal dystonia characterized by involuntary and often painful muscle contractions, leading to abnormal cervical movements and postures. While botulinum toxin injections are the first-line treatment, additional therapies, such as segmental muscle vibration (SMV), remain underexplored. SMV, a non-invasive neuromodulation technique, may enhance motor cortex excitability and promote neuroplasticity, offering potential benefits in PCD management. This single-center triple-blinded randomized controlled trial evaluates SMV’s efficacy in reducing dystonic pain and improving quality of life in PCD patients undergoing standardized rehabilitation after botulinum toxin treatment. Participants with a pain level of ≥3 on the Numerical Rating Scale will be randomized into two groups. The experimental group will receive 80 Hz SMV during a 10-session rehabilitation program, while the control group will undergo sham SMV. Both groups will follow identical physiotherapy and occupational therapy protocols. The primary outcomes include changes in pain intensity and function, assessed at baseline, mid-treatment, and post-treatment using validated scales. The secondary outcomes will evaluate quality of life and patient satisfaction. This study hypothesizes that SMV will significantly reduce dystonic pain and enhance quality of life, supporting its integration into multidisciplinary rehabilitation for dystonic disorders. Trial registration number: NCT06748846.

## 1. Introduction

Primary cervical dystonia (PCD) is the most prevalent form of focal dystonia, a neurological movement disorder characterized by sustained or intermittent muscle contractions causing abnormal, often repetitive movements or postures of the neck. The condition significantly impacts patients’ quality of life due to chronic pain, social embarrassment, and functional impairments. The global prevalence of PCD is estimated at 5 to 50 cases per 100,000 individuals, with variations attributed to diagnostic criteria and population studies. In Europe, the prevalence has been reported to range from 5.7 to 8.9 per 100,000 [1], while, in the United States, studies estimate approximately 30 to 50 cases per 100,000 adults [2]. Notably, PCD typically manifests in middle adulthood, with a slight female predominance. The clinical presentation of PCD is heterogeneous, with patients exhibiting abnormal head postures, such as torticollis (rotation), laterocollis (tilting), anterocollis (flexion), or retrocollis (extension). These abnormal postures are often accompanied by hypertrophy of the involved muscles and may be associated with tremor, pain, or both. The symptom severity varies widely, ranging from mild postural disturbances to severe and disabling deformities. The pathophysiology of PCD involves dysfunction in sensorimotor integration within the basal ganglia, cortex, and cerebellum, leading to altered muscle activation patterns [3]. Despite advances in neuroimaging and neurophysiological techniques, the precise mechanisms remain incompletely understood [4]. The current gold-standard treatment for PCD is botulinum toxin (BoNT) injections, which provide temporary relief by inhibiting acetylcholine release at the skeletal neuromuscular junction, thereby reducing muscle overactivity. BoNT has demonstrated to be safe and effective in improving pain, disability, and the severity of PCD [5,6]. However, the limitations include the need for repeated injections every 3–4 months, variable response, and potential side effects, such as dysphagia and muscle weakness. Adjunctive therapies, including oral medications such as anticholinergics, benzodiazepines, and deep brain stimulation (DBS) in refractory cases, offer alternative management strategies but are associated with their own limitations [3,7].

Segmental muscle vibration (SMV) is an emerging neuromuscular therapeutic modality that utilizes high-frequency mechanical vibrations applied directly to a specific muscle or muscle group using a handheld or fixed device. This modality of vibration administration enables localized and well-targeted application, in contrast to another well-known vibratile technique, namely whole-body vibration, which involves standing, sitting, or performing exercise and tasks on a vibrating platform, leading to global neuromuscular stimulation [8].

SMV evokes multifaceted neurophysiological responses that are primarily mediated through the activation of muscle spindle Ia afferents, with differential effects depending on the frequency, amplitude, and application site. The sensory effects involve preferential activation of primary muscle spindle afferents (with bag fibers strongly responding to 70–80 Hz stimulation [9]), Golgi organ tendons, particularly when the receptor-bearing muscle is tonically contracted [10], and cutaneous mechanoreceptors, with afferent signals propagating via the dorsal column–medial lemniscus pathway. The motor effects manifest as the tonic vibration reflex involving monosynaptic excitation of homonymous motor neurons, polysynaptic facilitation of synergist muscles, and reciprocal inhibition of antagonist muscles. Moreover, vibration has an inhibitory effect on the myotatic reflex [11]. The perceptive effects of SMV include kinesthetic illusions of limb movement and position sense distortion, resulting from central interpretation of abnormal proprioceptive input (i.e., stretching of the muscle) in the somatosensory and posterior parietal cortices [9].

The afferent stimulations of SMV are thought to enhance proprioceptive feedback, normalize sensorimotor integration, and induce neuroplastic changes, potentially restoring more physiological muscle activation patterns [12]. The neurophysiological rationale for SMV is grounded in its ability to influence the gamma motor system and alter cortical excitability. Studies have demonstrated that vibration can modulate somatosensory and motor cortex activity, reduce maladaptive plasticity, and improve motor function These effects have been explored in various neurological disorders, including stroke, Parkinson’s disease, and spasticity, where SMV has shown promise in improving motor control, reducing muscle tone, and enhancing functional outcomes [13]. In the context of dystonia, the preliminary evidence suggests that SMV may address the underlying sensorimotor integration deficits by recalibrating aberrant afferent input and modulating basal ganglia and cerebellar circuits. However, the application of SMV in PCD remains largely unexplored, with only a few case studies and small-scale trials investigating its efficacy as a non-invasive and well-tolerated therapeutic option. For instance, a proof-of-concept study demonstrated that vibrotactile stimulation of the neck reduced pain in individuals with dystonia [14]. Additionally, a case study exploring neck muscle vibrotactile stimulation as a treatment for cervical dystonia reported pain relief and improvements in electrophysiological and kinematic data [15]. These studies have reported improvements in head posture, pain, and functional disability, highlighting the potential of SMV as a non-invasive and well-tolerated therapeutic option.

Building on the pathophysiological understanding of PCD and the neurophysiological mechanisms of SMV, this randomized controlled trial (RCT) hypothesizes that SMV can improve clinical outcomes in patients with PCD by normalizing sensorimotor integration and reducing maladaptive muscle activation. Specifically, the objective of this study will be to assess the efficacy of SMV in adjunction to standard care in improving PCD symptoms, compared to sham SMV plus standard care, in terms of pain, cervical dystonia severity, disease-related disability, and patients’ self-perceived performance and satisfaction in daily activities.

By addressing this objective, this RCT seeks to establish SMV as a novel therapeutic approach for PCD, potentially expanding the treatment strategy of non-invasive interventions for this challenging condition. Moreover, the findings of this study may contribute to a deeper understanding of sensorimotor integration deficits in dystonia and pave the way for further research into vibration-based therapies in movement disorders.

## 2. Materials and Methods

### 2.1. Study Design and Setting

This is a protocol study for a randomized controlled trial with parallel arms. It will be conducted at the “E.Spalenza—Don Gnocchi” Rehabilitation Center in Rovato (BS), a tertiary referral rehabilitation center in Northern Italy. The center is equipped with specialized facilities and experienced staff to manage patients with neurological disorders, including cervical dystonia. The study is designed to include both experimental and control groups, with interventions administered in a controlled clinical environment.

### 2.2. Eligibility Criteria

Participants will be selected based on the following inclusion and exclusion criteria:

- Inclusion criteria
  1. Adults aged 18 years old or older, both male and female.
  2. Clinical diagnosis of primary cervical dystonia with reported pain intensity scores greater than 3 on a numeric rating scale for pain (from 0 to 10).
  3. Clinical stability at the time of enrollment.
  4. Ability to provide informed consent.
  5. No contraindications to botulin toxin type-A treatment.
- Exclusion criteria
  1. Diagnosis of generalized dystonia or non-cervical focal dystonia.
  2. Comorbid conditions such as neurodegenerative, neuromuscular, or uncontrolled inflammatory diseases.
  3. Cognitive impairment with Mini-Mental State Examination score < 24 or language and cultural barriers.
  4. Prior treatment with botulin toxin type-A within 30 days before the study.

### 2.3. Interventions and Procedure

**Figure 1:**
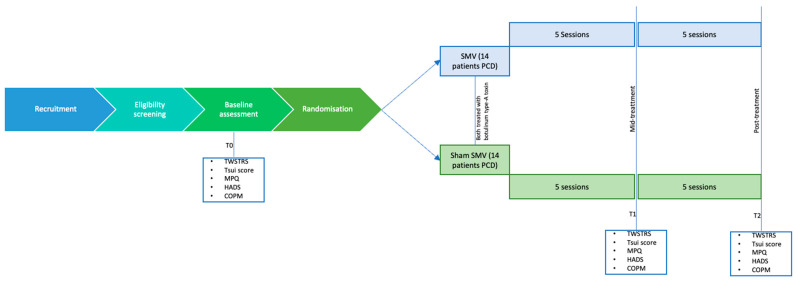
Flow chart of the study design.

Participants will be randomly divided into two groups: the experimental group (receiving SMV) and the control group (receiving sham SMV). All participants will receive standard treatment comprising botulin toxin type-A injections, physical therapy (PT), and occupational therapy (OT). Both groups will receive a total of 10 sessions of treatment in a span of 5 weeks (Figure 1).

#### 2.3.1. Segmental Muscle Vibration Intervention

**Figure 2:**
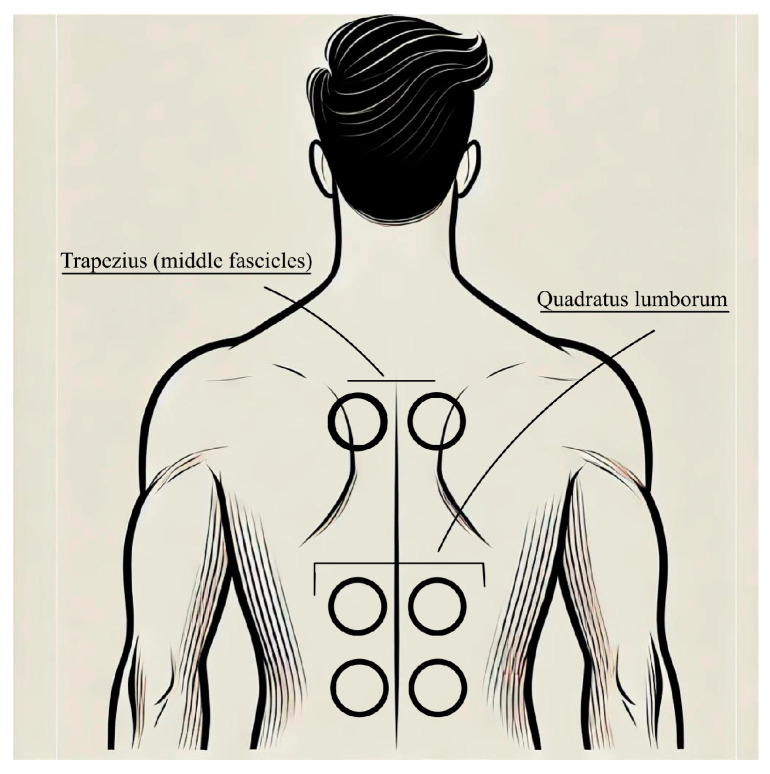
SMV probes’ positioning.

The SMV will be administered using the VIBRA Plus^®^ system, delivering mechano-sonic waves at a frequency of 80 Hz and a surface pressure of 210 mbar. Each session will last 15 min, conducted at the end of the PT session. The target muscles for vibration will be the descending and middle fascicles of the trapezius and the quadratus lumborum bilaterally (Figure 2). A vibratile probe is placed on each muscle and individually connected to the central device by a flexible plastic tube. The SMV is administered with the patient in a prone or sitting position. Participants in the sham group will receive treatment with the flexible plastic tube closed, ensuring no therapeutic effect. Rosenkranz [16,17] observed that, during stimulation periods of approximately 15 continuous minutes at frequencies close to 80 Hz, there is a decrease in what is referred to as short-interval intracortical inhibition. This results in an increased activation of cortical areas associated with the stimulated muscles, which, however, is counterbalanced by an enhancement in long-interval intracortical inhibition. This phenomenon appears to be related to modulation and alterations in cortical GABAergic inhibitory circuits [18], as well as the phenomenon known as Long-Term Potentiation, which underlies the mechanisms of synaptic plasticity [19].

#### 2.3.2. Physical Therapy and Occupational Therapy

The PT session will focus on proprioceptive and postural control, including exercises for muscle strengthening, mobilization, stretching, and postural adjustments. Each session will last 45 min. OT sessions, lasting 30 min, will concentrate on enhancing proprioceptive and praxis control to improve daily living activities. The physical therapy program will focus on improving proprioception, postural control, and muscle function to address the imbalances and discomfort caused by the condition. Sessions will integrate strengthening exercises targeting weakened muscle groups to counteract the effects of overactive or dystonic muscles. Isometric and isotonic exercises for the neck, upper back, and shoulder girdle will be emphasized to enhance stability and endurance. Stretching protocols will be implemented to reduce tightness in hypertonic muscles, such as the sternocleidomastoid, trapezius, and scalene muscles, improving the range of motion and overall flexibility. Mobilization techniques, both passive and active, will aim to restore movement in the cervical spine and associated joints, while postural training will guide patients in achieving neutral cervical alignment. This training will include feedback through mirrors and verbal cues, alongside exercises that promote coordination between the neck and thoracic regions to minimize compensatory movement patterns. Relaxation techniques, including diaphragmatic breathing and progressive muscle relaxation, will help to manage pain and muscle tension. The occupational therapy approach will prioritize restoring functional independence and adapting daily activities to accommodate the challenges posed by cervical dystonia. Activity modification will be central to the intervention, with ergonomic adjustments to workspaces, tools, and tasks to reduce strain on the neck and upper body. Functional strengthening and coordination exercises will integrate task-specific movements, such as reaching, lifting, and fine motor tasks, like writing, to rebuild lost capabilities. Eye–hand coordination will also be a focus, using visual tracking and head positioning tasks to improve functional control.

### 2.4. Outcomes and Timepoints

The primary objective will be assessed using the following scales:

- The *Toronto Western Spasmodic Torticollis* Rating Scale (TWSTRS) is a widely recognized tool for assessing cervical dystonia. This scale evaluates the disorder across three distinct dimensions: severity, disability, and pain. The severity component measures the extent of abnormal postures, involuntary movements, and muscle hypertrophy, assessing the involvement of the neck and head in terms of rotation, tilt, flexion, or extension. The disability component examines the impact of the condition on daily activities, such as work, social interactions, and household tasks, highlighting the functional limitations caused by dystonia. Lastly, the pain dimension focuses on the intensity, frequency, and duration of pain, as well as its association with dystonic postures. Each dimension is scored independently, and the cumulative score provides a comprehensive picture of the condition’s burden, making the TWSTRS an essential tool for both clinical practice and research in cervical dystonia [20].
- The *Tsui score* is a clinical rating scale used to assess the severity of cervical dystonia. It provides a quantitative evaluation of the disorder by examining several aspects of the condition, including the type and extent of abnormal neck movements, sustained postures, and tremor. The scoring system considers the degree of head deviation in different planes (e.g., rotation, tilt, or flexion–extension), the presence of sustained postures, and the frequency and intensity of dystonic tremor. Additional modifiers are applied for factors such as shoulder elevation or hypertrophy. The Tsui score offers a comprehensive yet concise evaluation of cervical dystonia, making it a valuable tool for tracking disease progression and evaluating treatment efficacy in both clinical and research settings [21].
- The *McGill Pain Questionnaire* (MPQ) is a multidimensional tool designed to assess the complex nature of pain by evaluating its sensory, affective, and evaluative components. This questionnaire provides a comprehensive framework for understanding how pain is experienced and perceived by individuals. The sensory dimension focuses on the physical qualities of pain, such as its intensity, location, and specific characteristics like sharpness, burning, throbbing, or stabbing. The affective dimension explores the emotional aspects of pain, such as distress, anxiety, or unpleasantness, evaluating how pain impacts the individual’s mood and emotional well-being. The evaluative dimension assesses the overall experience of pain by considering its severity and the individual’s subjective perception of its impact on daily life. The MPQ is widely used in clinical and research settings for its ability to provide a detailed understanding of pain, making it useful for tailoring pain management strategies [22].
- The *Canadian Occupational Performance Measure* (COPM) is a client-centered tool used to evaluate an individual’s self-perceived performance and satisfaction in daily activities, focusing on self-care, productivity, and leisure. Patients identify and prioritize meaningful activities, rate their importance (1–10), and assess their performance and satisfaction levels (1–10). Administered at baseline and post-intervention, the COPM measures changes over time, helping to evaluate treatment effectiveness and guide personalized care. Widely used in rehabilitation, it emphasizes patient collaboration and relevance to individual goals [23,24]

Assessments will be conducted at baseline (T0), mid-treatment after 5 sessions (T1), and post-treatment at the end of the 10th session (T2). The COPM will not be repeated at T1 due to its sensitivity limitations in mid-course evaluations.

### 2.5. Sample Size

The sample size was determined based on the primary outcome TWSTRS, referring to previous studies [25,26]. The sample size was calculated using G*Power 3.1 software, considering an effect size (d) according to Cohen’s “large” (d = 0.8), two degrees of freedom with a power (1-beta) of 80%, and alpha of 5%. Considering a drop-out rate of 20% resulted in a sample size of 14 subjects per group, for a total of 28 subjects.

### 2.6. Allocation, Randomization, and Blinding

Randomization will be conducted using sealed opaque envelopes prepared by an independent researcher. Each participant will be assigned to either the experimental or sham/control group in a 1:1 ratio, ensuring unbiased allocation. The study will employ a triple-blind design. Participants will be blinded to their group allocation (experimental or sham). The therapists administering the interventions will not be blinded due to the nature of the treatment but will not be involved in outcome assessments.

### 2.7. Statistical Methods

Descriptive statistics will summarize baseline characteristics and outcomes. Continuous variables will be presented as means ± standard deviations and categorical variables as frequencies and percentages. Inferential statistics will include paired *t*-tests or Wilcoxon signed-rank tests for within-group comparisons and independent *t*-tests or Mann–Whitney U tests for between-group comparisons. Statistical significance will be set at *p* < 0.05.

## 3. Conclusions

In conclusion, this randomized controlled trial aims to evaluate the efficacy and safety of segmental muscle vibration as an adjunctive therapy for managing primary cervical dystonia. By exploring its potential to alleviate pain and improve functional outcomes through neuromodulation and neuroplasticity, the study may pave the way for incorporating SMV into multidisciplinary rehabilitation protocols for dystonia. This innovative approach has the potential to expand non-invasive treatment strategies and enhance the quality of life for individuals with PCD.

## 4. Ethics and Dissemination

The protocol is created and presented in accordance with the Standard Protocol Items: Recommendations for Interventional Trials (SPIRIT) [27]. The study protocol (ID Studio: 10_29/06/2022) and informed consent documents have been reviewed and approved by the ethics committee of IRCCS Fondazione Don Carlo Gnocchi. If there is any amendment to the protocol, approval must be sought again from the Ethics Committee. The study strategy has been registered on ClinicalTrialGov (Identifier: NCT06748846), and the trial will be conducted following the principles of the Declaration of Helsinki and Good Clinical Practice guidelines.
